# Effects of Stripe Rust Infection on the Levels of Redox Balance and Photosynthetic Capacities in Wheat

**DOI:** 10.3390/ijms21010268

**Published:** 2019-12-31

**Authors:** Yanger Chen, Haotian Mao, Nan Wu, Jie Ma, Ming Yuan, Zhongwei Zhang, Shu Yuan, Huaiyu Zhang

**Affiliations:** 1 College of Life Science, Sichuan Agricultural University, Ya’an 625014, China; sicaumao@163.com (H.M.); sicaunanwu@163.com (N.W.); sicaujiema@163.com (J.M.); yuanming@sicau.edu.cn (M.Y.); 2 College of Resources, Sichuan Agricultural University, Chengdu 611130, China; zzwzhang@126.com (Z.Z.); roundtree318@hotmail.com (S.Y.)

**Keywords:** stripe rust, antioxidant enzyme, chlorophyll fluorescence, photosystem, *Triticum aestivum* L.

## Abstract

Wheat stripe rust (*Puccinia striiformis* f. sp. *tritici*, *Pst*) is the most destructive wheat disease and a major problem for the productivity of wheat in the world. To obtain a better understanding about different effects of redox homeostasis and photosystem (PS) to *Pst* infection in wheat, we investigated the differences in photosynthesis and the antioxidant defense system in wheat cultivar Chuanmai42 (CM42) in response to two Chinese *Pst* races known as CYR32 and V26. The results showed that V26-infected wheat accumulated a higher reactive oxygen species (ROS), cell death, and energy dissipation than CYR32-infected wheat when compared with the control. Furthermore, we found that the activities of three antioxidant enzymes (APX, GR, and GPX) and four resistance-related enzymes in CYR32-infected wheat were significantly higher than that in V26-infected wheat. In addition, quantitative RT-PCR indicated that the expression levels of two genes associated with resistant stripe rust in CYR32-infected wheat were clearly higher than that in V26-infected wheat. Compared with CYR32-infected wheat, lower photochemical efficiencies were observed in V26-infected wheat at the adult stage. Meanwhile, only a marked decline in D1 protein was observed in V26-infected wheat. We therefore deduced that wheat with stripe rust resistance could maintain high resistance and photosynthetic capacity by regulating the antioxidant system, disease-resistant related enzymes and genes, and the levels of PSII reaction center proteins.

## 1. Introduction

As one of the most important sources of calories for humans, wheat (*Triticum aestivum* L.) is a widely planted cereal crop in the world. However, the grain quality and yield of wheat usually are influenced by abiotic and biotic stresses in the field. Among various fungal diseases, stripe rust, also called yellow rust, is one of the most important wheat diseases on cereal crops and grasses worldwide. Wheat stripe rust is caused by Puccinia striiformis f. sp. tritici (*Pst*), which is a widespread disease, and can greatly reduce or even destroy wheat yield at an epidemic level [1,2]. Obtaining stripe rust resistant wheat cultivars are the most economical, effective, and environmentally-friendly method to control or decrease the damage by the disease [1,3]. However, wheat cultivars with race-specific resistance tend to lose stripe rust resistance and become susceptible to stripe rust because of the rapid evolution of new races [4]. Therefore, it is necessary to explore the mechanism of disease resistance and develop new strategies to improve disease resistance in wheat.

It has been reported that stripe rust is specially threatening in wheat growing regions in the Northern, Southwestern, and Northwestern China [1,5]. The major wheat growing regions along the Huai and Yellow river regions are usually the main places damaged by stripe rust. Previous studies indicated that *Pst* races including CYR32 and CYR33 are widely distributed in China and constitute the primary cause of wheat stripe rust [1]. It is reported that there are more than 80 stripe rust resistance genes in wheat [6]. As a stripe rust resistance gene, *Yr26*/*24* has a high resistance to the main *Pst* race CYR32 and has been used to identify resistant wheat cultivars such as Chuanmai42 (CM42) and Guinong 22 [5]. However, some previous reports indicated that the *Pst* race Yr26 (V26) is virulent on wheat cultivar CM42 in the Sichuan Basin [7,8]. At present, wheat resistance to stripe rust is usually categorized into all-stage resistance (also named seedling resistance) and adult-plant resistance (APR). Although all-stage resistance provides a higher resistance level as race-specific, it is easily overcome by new virulent races [1]. Wheat cultivar CM42 is an elite wheat germplasm with high all-stage resistance to CYR32 and all-stage susceptibility to V26 [9], while the mechanisms of a different level of stripe rust resistance are still unknown in CM42.

It is well known that the reactive oxygen species (ROS) are usually accumulated in plants under biotic stresses [10]. Many research studies also have indicated that a series of defense responses are involved in all-stage resistance against *Pst* infection in wheat [11,12,13], mainly including the production of ROS, papilla formation, and cell wall apposition. However, ROS generation and scavenging have been shown to depend on the interactions between plants and pathogen. Our previous study indicated that the susceptible wheat in response to stripe rust disease accumulated higher levels of ROS than the resistant wheat cultivars at APR [14]. However, wheat plants have involved an accurate mechanism to defend themselves from the ROS attack by an efficient antioxidant defense system including antioxidant enzymes and antioxidant metabolites when it is exposed to stripe rust infection [14,15]. Therefore, the levels of ROS accumulation and the antioxidant defense system are closely related to stripe rust resistance in wheat [14,16].

Photosynthesis, as the most important chemical reactions on Earth, has been thought to be involved in plant yield [17]. However, photosynthesis is usually impacted by different biotic and abiotic stresses. It is well known that *Pst* infection can change the photosynthetic efficiency of the host wheat. Some studies indicated that the photosynthetic activities were closely correlated with stripe rust, and PSII was easily damaged by *Pst* infection in wheat [14,18]. Even though in a resistant wheat cultivar, the photosynthetic rate significantly decreased after symptoms appear and signs develop. Similarly, a significant decline in the net photosynthetic rate was observed in compatible interactions. Although some previous studies have demonstrated that stripe rust infection may lead to the decline in photosynthetic capacities, the mechanism by which the PSII responds to stripe rust between the compatible and the incompatible interactions are poorly understood. 

In the present study, we investigated the changes in the levels of ROS metabolism and photosynthesis in stripe rust infected wheat. In particular, several resistant-related enzymes and genes in response to wheat stripe rust were examined. Our results indicate that the antioxidant system, disease-resistant enzymes, and PSII reaction center proteins are associated with the response to stripe rust in wheat. In this case, we aim to elucidate wheat resistance mechanisms against stripe rust infections, and, thus, to improve the stripe rust resistance and the yield of wheat.

## 2. Results

### 2.1. Symptoms of Wheat Leaves Infected with Pst

**Figure 1:**
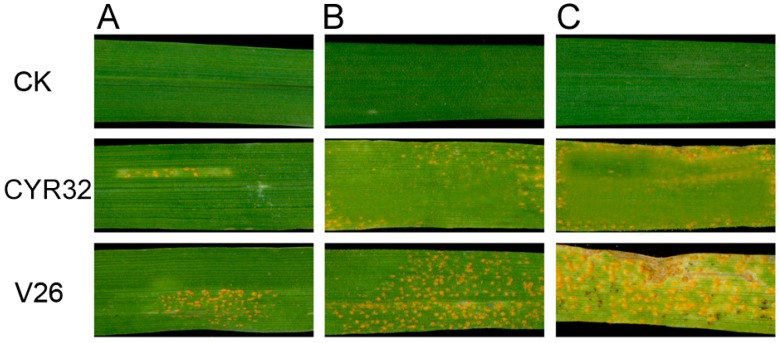
Symptomatology of wheat cultivar Chuanmai42 (CM42) inoculated with CYR32 and V26. A-C represent different day-after inoculation with *Pst*. CK, un-inoculated wheat leaves.

To investigate stripe rust resistance at all stages, the symptoms of CM42 leaves infected with CYR32 and V26 were observed after different days of inoculation (Figure 1). No clear disease symptom appeared on mock-inoculated leaves. However, the leaves showed high susceptibility with clear stripe rust uredospore after different days post-inoculation with V26. In contrast, there were very few spores at the edges of short necrotic stripes on the leaves of CM42 after inoculation with race CYR32. Therefore, our results demonstrated that CM42 is resistant to race CYR32 and susceptible to race V26 at all stages.

### 2.2. Changes in Chlorophyll (Chl) Content, Total Protein Content, and Osmotic Regulators

The contents of Chl, total protein, soluble sugar, and proline showed no clear differences in mock-inoculated plants at 24, 48, and 120 h post-inoculation (hpi) (Figure S1). Compared with the control, total Chl content significantly decreased by 12.5% and 29.2% at 120 hpi with CYR32 and V26, respectively (Figure S1A). However, there was no significant decrease in the content of total protein after inoculation of CYR32 and V26 compared with the control (Figure S1B). Furthermore, the contents of two osmotic regulators (proline and soluble sugar) were determined in CM42 leaves infected with *Pst*. Compared with the control, soluble sugar and proline content significantly increased at 24, 48, and 120 hpi in the compatible and incompatible systems (Figure S1C,D). However, a more marked increase in soluble sugar and proline were found in the compatible system compared with the incompatible system.

### 2.3. Pst Infection Induced Oxidative Damage and Cell Death

**Figure 2:**
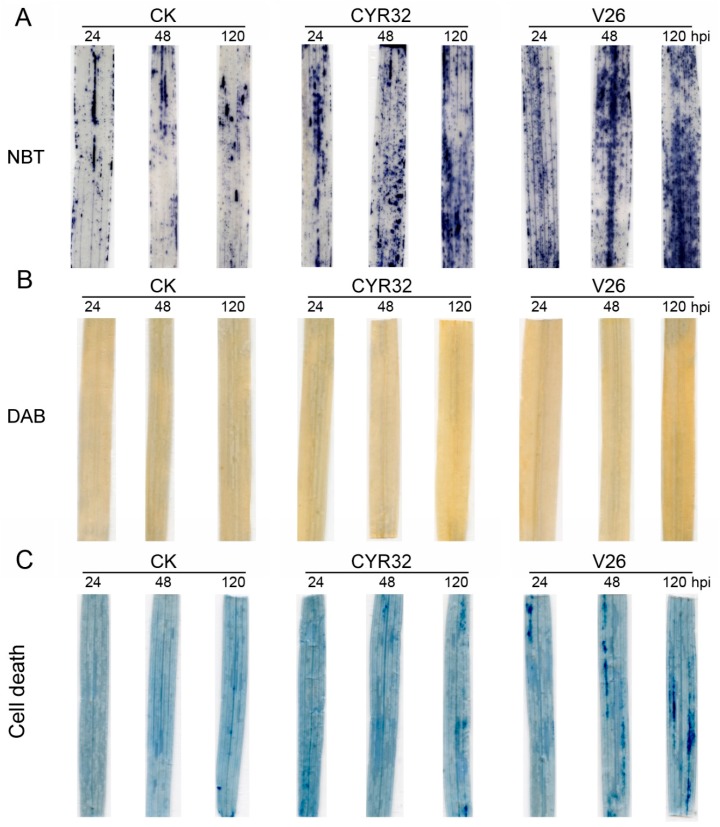
Measurement of reactive oxygen species (ROS) and cell death in wheat cultivar CM42 inoculated with CYR32 and V26. Histochemical analysis for superoxide anion radicals (O_2_˙ˉ) and hydrogen peroxide (H_2_O_2_) by nitro blue tetrazolium (NBT) (**A**) and 3,3-diaminobenzidine (DAB) (**B**) staining, respectively. (**C**) Degree of cell death was visually detected by trypan blue staining. CK, un-inoculated wheat plants. 24–120 hpi represent 24, 48, and 120 h post-inoculation.

To explore the oxidative damage to *Pst* infection between the compatible and incompatible systems, the levels of ROS mainly including H_2_O_2_ and O_2_˙ˉ were analyzed in CM42 inoculated with CYR32 and V26. Histochemical staining of ROS showed that the levels of O_2_˙ˉand H_2_O_2_ in the compatible and incompatible systems significantly increased after inoculation compared with the control (Figure 2A,B), especially for 120 hpi. However, relative to the incompatible system, the compatible system presented higher accumulation of O_2_˙ˉ and H_2_O_2_ in CM42, especially for O_2_˙ˉ at 48 and 120 hpi. To confirm these results obtained from histochemical staining, the rate of O_2_˙ˉ production and H_2_O_2_ content were further measured in CM42 inoculated with CYR32 and V26. Compared with the control, infection with CYR32 and V26 resulted in the significant increase in the rate of O_2_˙ˉ production and H_2_O_2_ content at 48 and 120 hpi (Figure S2A,B). Similarly, a higher O_2_˙ˉ production rate and H_2_O_2_ content were found in the compatible system relative to the incompatible system. It was well known that malondialdehyde (MDA) and electrolyte leakage were regarded as the important index in evaluating the lipid peroxidation [14]. As shown in Figure S2C,D, a sharp increase in MDA content and electrolyte leakage was observed in the compatible and incompatible systems, especially for 120 hpi, which was consistent with the results from ROS accumulation. These results demonstrated that CM42 infected with V26 suffered from more severe oxidative damage.

**Figure 3:**
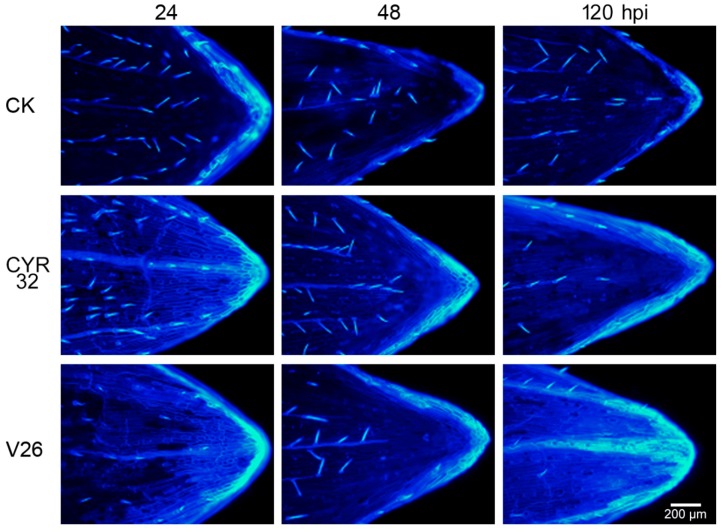
Callose deposition of wheat cultivar CM42 inoculated with CYR32 and V26. The un-inoculated and inoculated leaves were stained with aniline blue at 24, 48, and 120 hpi. The images are observed with the fluorescence microscope. Bars = 200 µm. CK, un-inoculated wheat plants. 24–120 hpi represent 24, 48, and 120 h post-inoculation.

ROS are thought to be an important stress signal in the regulation of the programmed cell death pathway [19]. To observe the degree of cell death in CM42 infected with CYR32 and V26, trypan-blue staining was carried out at 24, 48, and 120 hpi. Compared with the control, cell death was found in the compatible system, especially for 48 and 120 hpi (Figure 2C). However, the incompatible system presented a slighter cell death than the compatible system at all stages after inoculation. In addition, CM42 infected with V26 showed higher callose deposition compared with an infection by CYR32 (Figure 3). Therefore, these results also indicated that the compatible system suffered from more serious damage infected by strip rust.

### 2.4. Effects of Stripe Rust on the Antioxidant System, Disease-Resistant Related Enzymes, and Genes

**Figure 4:**
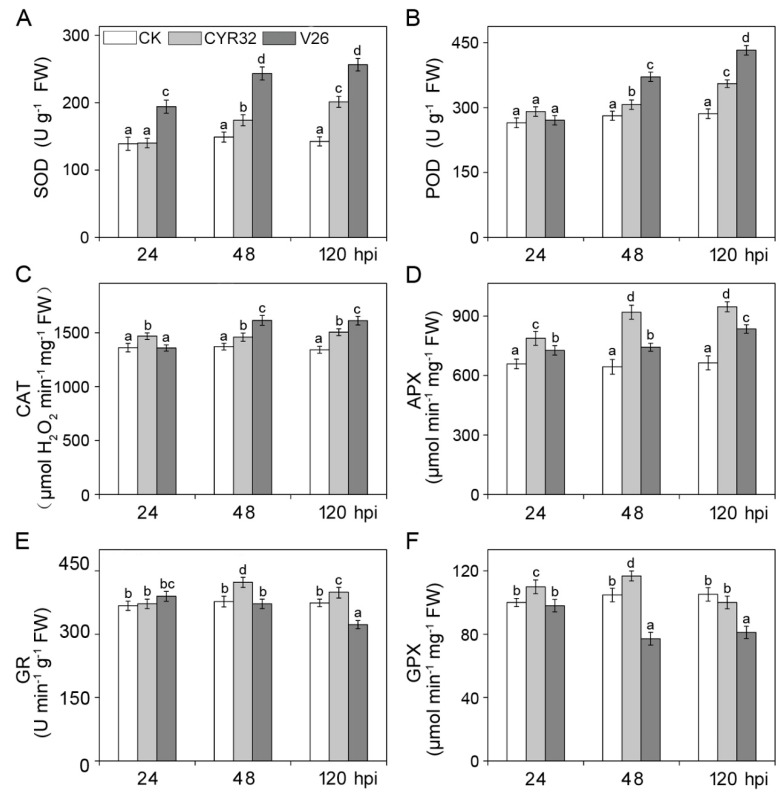
The activities of six antioxidant enzymes in wheat cultivar CM42 inoculated with CYR32 and V26. SOD, superoxide dismutase (**A**). POD, peroxidase (**B**). CAT, catalase (**C**). APX, ascorbate peroxidase (**D**). GR, glutathione reductase (**E**). GPX, glutathione peroxidase (**F**). Bars represent standard deviations (SD), which were calculated from four independent biological replicates (*n* = 4). Different letters note significant differences among different treatments (*p* < 0.05) following Duncan’s multiplication range test. CK, un-inoculated wheat plants. 24–120 hpi represent 24, 48, and 120 h after inoculation.

To investigate the effect of stripe rust on the antioxidant defense system in the compatible and incompatible systems, the activities of antioxidant enzymes and the concentrations of antioxidant metabolites were measured after infection by *Pst*. As shown in Figure 4, the antioxidant enzyme activities in the un-inoculated plants showed no significant difference at 24, 48, and 120 hpi. Compared with the control, the activities of six antioxidant enzymes significantly increased in the incompatible system at 48 and 120 hpi (Figure 4). However, the activities of six antioxidant enzymes showed different changes in the compatible system after inoculation with V26. Compared with the control, the activities of superoxide dismutase (SOD), peroxidase (POD), catalase (CAT), and ascorbate peroxidase (APX) presented the significant increase in the compatible system (Figure 4A–D), especially at 120 hpi. In contrast, the pronounced decline in the activities of glutathione reductase (GR) and glutathione peroxidase (GPX) were observed in CM42 infected with V26 relative to the control plants (Figure 4E,F). Moreover, the contents of dehydroascorbate (DHA) and oxidized glutathione (GSSG) significantly increased, while the concentrations of reduced ascorbic acid (AsA) and reduced glutathione (GSH) remarkably decreased in the compatible and incompatible systems (Figure S3). However, a more significant decline or increase in the contents of antioxidant metabolites were observed in the compatible system relative to the incompatible system.

**Figure 5:**
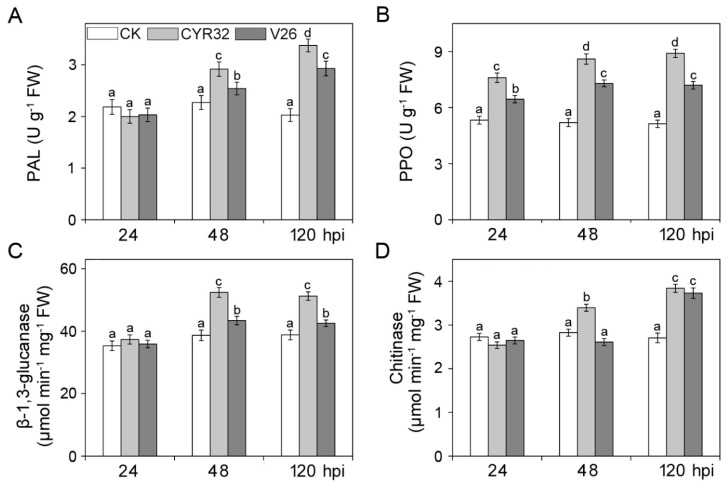
The activities of PAL, phenylalanine ammonia lyase (**A**). PPO, polyphenol oxidase (**B**). *β*-1,3-glucanase (**C**) and chitinase (**D**) in wheat cultivar CM42 inoculated with CYR32 and V26. Bars represent standard deviations (SD), which were calculated from four independent biological replicates (*n* = 4). Different letters note significant differences among different treatments (*p* < 0.05) following Duncan’s multiplication range test. CK, un-inoculated wheat plants. 24–120 hpi represent 24, 48, and 120 h post-inoculation.

**Figure 6:**
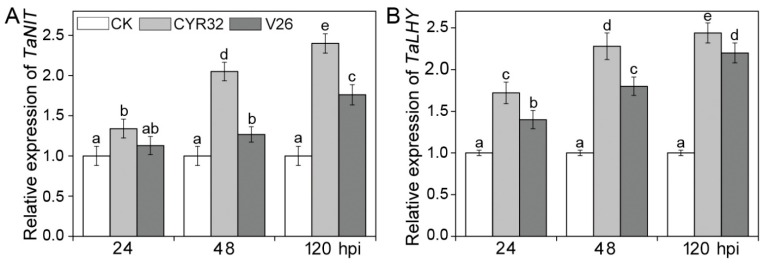
Expression profiles of *TaNIT* (**A**) and *TaLHY* (**B**) in wheat cultivar CM42 inoculated with CYR32 and V26. The *TaActin* gene was used as a positive control. The relative expression of two genes was calculated by using the comparative threshold (2^−ΔΔ*C*t^) method. Bars represent standard deviations (SD), which were calculated from four independent biological replicates (*n* = 4). Different letters note significant differences among different treatments (*p* < 0.05) following Duncan’s multiplication range test. CK, un-inoculated wheat plants. 24–120 hpi represent 24, 48, and 120 h post-inoculation.

To test the changes in disease-resistant related enzymes after *Pst* infection in the compatible and incompatible systems, the activities of PAL, PPO, *β*-1,3-glucanase, and chitinase were measured (Figure 5). The activities of four disease-related enzymes did not differ significantly in uninfected CM42 at three different stages. Compared with the uninfected controls, PAL, PPO, *β*-1,3-glucanase, and chitinase activities were dramatically elevated at 48 and 120 hpi in the compatible and incompatible systems (Figure 5). When compared with the compatible system, the incompatible system showed higher activities of four disease-related enzymes at 48 and 120 hpi. To further explore the molecular mechanisms of disease resistance, two important disease-resistant genes were investigated in CM42 infected by CYR32 and V26 (Figure 6). In mock-inoculated plants, there were no significant differences in the expression levels of *TaNIT* and *TaLHY* genes. Compared with mock-inoculated plants, the expressions of *TaNIT* and *TaLHY* in both the compatible and incompatible systems were significantly up-regulated at 24, 48, and 120 hpi. However, inoculation with CYR32 led to higher expression of *TaNIT* and *TaLHY* than that of inoculation with V26 at three stages. Therefore, these results indicated that disease-related enzymes and disease-resistant genes play the important regulatory roles in the incompatible system.

### 2.5. Effects of Wheat Stripe Rust on Photosynthetic Capacities

**Figure 7:**
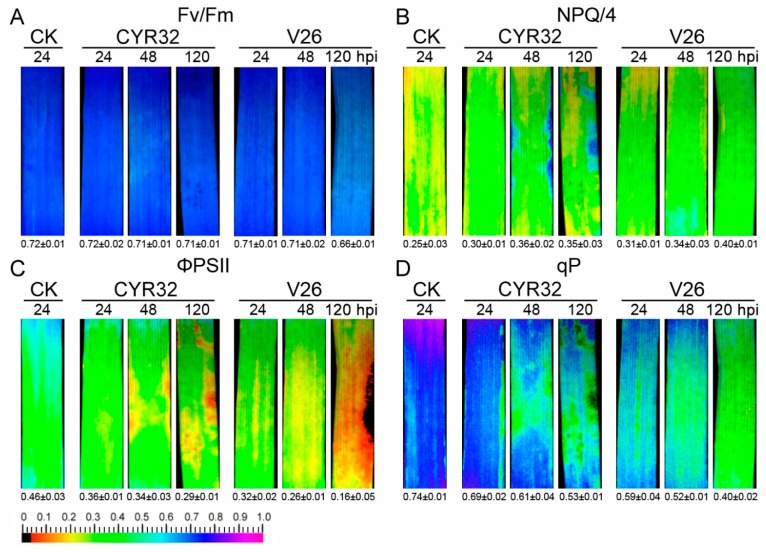
Chlorophyll fluorescence parameters in wheat cultivar CM42 inoculated with CYR32 and V26. Fv/Fm, maximum efficiency of PSII photochemistry (**A**). NPQ, non-photochemical quenching (**B**). ΦPSII, quantum yield of PSII electron transport (**C**). qP, photochemical quenching (**D**). Quantitative values (± SD) are shown below each fluorescence images. CK, un-inoculated wheat plants. 24–120 hpi represent 24, 48, and 120 h post-inoculation.

**Figure 8:**
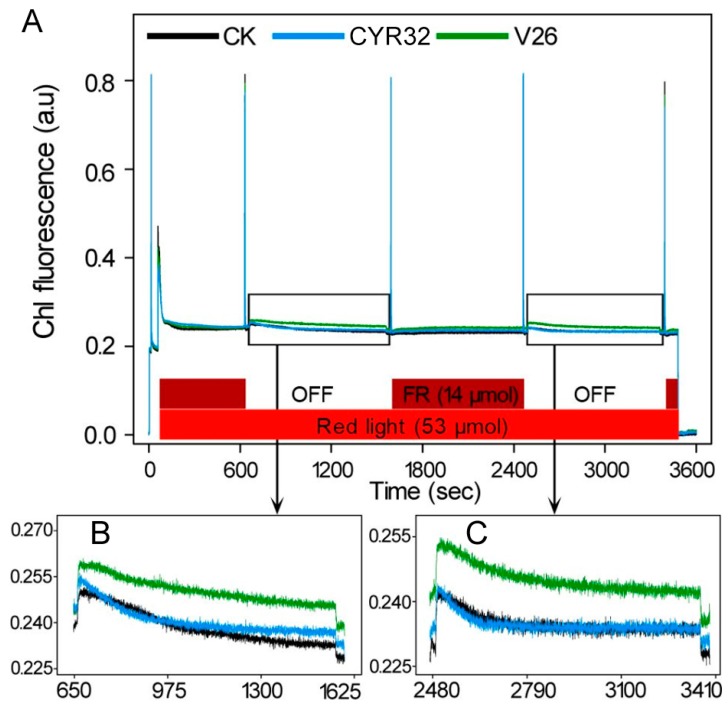
Assays of state transitions in wheat cultivar CM42 inoculated with CYR32 and V26 at 120 hpi. (**A**) Pulse amplitude-modulated fluorescence traces after shifts from state 1 to state 2 light and back. (**B**,**C**) Enlargement of a section of (**A**). Fluorescence response curves when illumination of light is changed back to favor PSII (state 1 to state 2 transition). The bars at the bottom show illumination with red (shown in red) and far-red (dark red) light. Fluorescence is shown in arbitrary units.

To investigate whether the incompatible system had high photosynthetic capacity after inoculation, PSI and PSII photochemistry of the leaves infected by CYR32 and V26 was examined by a modulated fluorometer. No significant differences in maximum efficiency of PSII photochemistry (Fv/Fm), non-photochemical quenching (NPQ), quantum yield of PSII electron transport (ΦPSII), and photochemical quenching (qP) were observed in the mocked-inoculated plants at 24, 48, and 120 hpi (Figure S4). Compared with the control, a significant decrease in Fv/Fm was found in the compatible system at 120 hpi (Figure 7A). Furthermore, we found that the value of NPQ was remarkably increased in the compatible and incompatible systems at 48 and 120 hpi (Figure 7B). However, ΦPSII and qP values were significantly decreased in the compatible and incompatible systems compared with the mock-inoculated plants (Figure 7). When compared with the incompatible system, a more clear decrease in ΦPSII and qP were found in the compatible system at 48 and 120 hpi. In addition, we also detected the capacity of state I to state II transition. As shown in Figure 8A, the balance of excitation between PSI and PSII in the compatible and incompatible systems was not similar when compared with the control. A detailed investigation of PSII fluorescence traces showed that the absorption of light in PSII resulted in a greater increase in PSII fluorescence in the compatible system than in the mock-inoculated plants (Figure 8B,C). However, PSII fluorescence showed no clear difference between the control plants and the incompatible system (Figure 8).

To further explore photosynthetic capacity in the compatible and incompatible systems, the maximal P700 signal (*P*m) was determined in CM42 infected by CYR32 and V26. PSI quantum yield (ΦPSI) in the compatible and incompatible systems was similar with that of the mock-inoculated plants under moderate and high light intensities (Figure S5A). Under moderate and high light intensities, there were significant increases in the quantum yield of non-photochemical energy dissipation of PSI reaction centers due to acceptor side limitation (Φ_NA_) and decrease in the quantum yield of non-photochemical energy dissipation in PSI reaction centers due to donor-side limitation (Φ_ND_) in the compatible and incompatible systems relative to the control (Figures S5B and S6C). However, the remarkable decrease in *P*m only occurred at 120 hpi in the compatible and incompatible systems (Figure S5D). Compared with the incompatible system, the compatible system presented a remarkable decline in PSI photochemistry at 48 and 120 hpi. These results indicated that V26 infection reduced the linear electron transport of PSI in CM42.

Next, four gas exchange parameters in the inoculated and non-inoculated CM42 were analyzed. Compared with the control, V26 infection caused the significant decrease in net photosynthetic rate (*P*n), transpiration rates (*T*r), intercellular CO_2_ concentration (*C*i), and stomatal conductance (*G*s) in CM42 at 24, 48, and 120 hpi (Figure S6). However, a significant decline in *P*n, *T*r, *C*i, and *G*s was found at 120 hpi in the incompatible system. Therefore, these findings suggested that V26 infection resulted in the clear decline in photosynthetic efficiency in CM42.

### 2.6. Effects of Stripe Rust on Thylakoid Membrane Proteins

**Figure 9:**
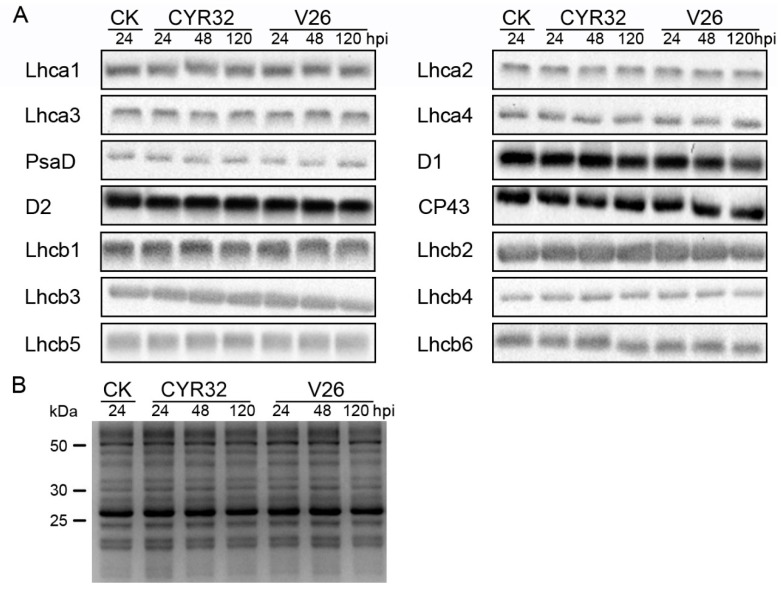
Immunoblot analyses of thylakoid proteins obtained from the un-inoculated and inoculated wheat plants. (**A**) Immunoblotting of thylakoid proteins were done using specific antibodies against representative photosystem I (PSI) proteins (Lhca1, Lhca2, Lhca3, Lhca4, and PsaD) and photosystem II (PSII) proteins (D1, D2, CP43, Lhcb1, Lhcb2, Lhcb3, Lhcb4, Lhcb5, and Lhcb6). (**B**) The SDS-PAGE results after Coomassie Brilliant Blue staining (CBS) are shown in the bottom panel. Loading was based on an equal amount of chlorophyll (1 μg Chl) into each electrophoretic lane. CK, un-inoculated wheat plants.

To determine whether a reduction in the abundance of photosynthetic proteins occurred after inoculation with CYR32 and V26, thylakoid polypeptide composition was analyzed by immunoblotting (Figure 9). There was no significant difference in the amount of thylakoid proteins in the control plants at 24, 48, and 120 hpi. Compared with the control, the contents of almost all the analyzed thylakoid membrane proteins showed no detectable changes in the compatible and incompatible systems at 24, 48, and 120 hpi. Only the level of D1 protein was markedly reduced in the compatible system at 48 and 120 hpi compared with the control, especially at 120 hpi (Figure 9A). Therefore, these results indicated that *Pst* infection did not clearly influence the levels of photosynthetic proteins except for a PSII reaction center protein.

## 3. Discussion

It has been known that wheat stripe rust can cause devastating diseases in the world. It is important to investigate the response to different *Pst* infection in the same wheat cultivars. To better understand the detailed mechanisms of the oxidative damage and photosynthesis decline in the compatible and incompatible systems, we studied the changes in photosynthetic characteristics and redox equilibrium in CM42 infected by CYR32 and V26.

The decline in photosynthetic pigments is a very common phenomenon under abiotic and biotic stresses [14,20]. Many previous reports have indicated that Chl contents decrease in rust-infected cereal tissues [14,16]. Our previous study also demonstrated that stripe rust infection led to a higher decline in the amount of Chl in susceptible wheat relative to the resistant wheat [14]. Consistent with these previous findings, the present experiment showed that a more remarkable reduction in Chl content occurred in the compatible system. The reason may be because CM42 could provide a more effective protective response to CYR32 infection by alleviating the damages to pigments in the incompatible system. In addition, soluble sugar and proline are thought to be the two most important organic solutes in response to different stresses in plants [21,22]. Sugar consistently plays an important role between pathogen and host interactions. Some previous studies indicated that sucrose accumulation was observed in *Pst* infected wheat as well as in barley infected by powdery mildew [23,24]. In the present study, we found that proline and soluble sugar significantly increased in both the CYR32-infected and V26-infected CM42 leaves, which suggests that proline and sugar is involved in the response to *Pst* infection in wheat. In the compatible and incompatible systems, the different increase in soluble sugar was likely because sucrose plays different roles in these two systems. Previous studies indicated that sucrose may act as the source of carbon and energy needs for fungal growth in the compatible system [25,26] or as a signaling molecule in regulating the defense response to *Pst* infection in the incompatible system [27,28,29].

ROS are accumulated in different cellular compartments of plants in response to abiotic and biotic stresses. Furthermore, ROS accumulation has been reported in plants infected with various pathogens and is considered to be involved with the resistance to wheat stripe rust [30,31,32,33]. A previous report showed that a rapid ROS generation after *Pst* infection was observed in an incompatible interaction [14]. In contrast, ROS accumulation is lower in a compatible system compared to the incompatible interaction [16]. However, in the present experiment, we found that the accumulation of ROS was more pronounced in the compatible system relative to the incompatible system. The results indicated that the incompatible system can scavenge the excessive ROS effectively because a low concentration of ROS can induce protective antioxidant mechanisms and activates signaling pathways [19,34]. In the compatible system, high levels of ROS should be toxic to pathogens and, subsequently, trigger programmed cell death (PCD) [16,19,35]. These findings were consistent with our previous study, which indicated that the resistant wheat accumulated a lower level of ROS compared with the susceptible wheat after infection by *Pst* [14]. Furthermore, ROS accumulation can cause lipid peroxidation under environmental stress, which will lead to the oxidative damage of the cell membrane [36]. The present study showed that the compatible system had a high lipid peroxidation after inoculation, which was in accordance with the results from ROS accumulation. In addition, these results were further confirmed by the experiments of cell death and callose deposition, which are one of the multiple defense responses involved in wheat all-stage resistance against stripe rust infection [13]. Previous studies have indicated that callose deposition likely plays a key role in limiting colonization and, subsequently, prevent sporulation in some plants [37,38]. Similarly, we found that wheat plants promote a defense response through callose deposition after *Pst* infection, especially in the compatible system.

It has been well known that plants can alleviate or eliminate oxidative damage via a complex antioxidant defense system under different environmental stresses [39]. Antioxidant defense is likely a common strategy for stripe rust to suppress host basal immunity. Some previous reports indicated an antioxidant system may play an important role in regulating ROS accumulation during plant-pathogen interactions [14,16,40]. In this study, six antioxidant enzymes maintained high activities in CM42 infected by CYR32, which suggests that wheat plants can reduce ROS accumulation in the incompatible system. In the compatible system, high activities of SOD, POD, and CAT were likely because these three antioxidant enzymes might play a role in ROS scavenging after infection by V26. In contrast, the remarkable decline in GR and GPX activities in the compatible system may be due to the severe oxidative damage to wheat plants. In addition, AsA is the important substrate of the AsA-GSH cycle due to its metabolic and antioxidative functions [41]. A previous report indicated that H_2_O_2_ accumulation was increased and AsA content was reduced in the *TaMDHAR*-knockdown leaves infected by *Pst* [42]. This finding aligned with our results, in which the contents of AsA and GSH decreases, while the concentrations of DHA and GSSG were increased in infected wheat plants.

It is well-known that some disease resistance-related enzymes including phenylalanine ammonia-lyase (PAL), polyphenoloxidase (PPO), *β*-1,3-glucanase, and chitinase have been proven to play an important role in plant disease resistance against pathogen infection [42,43]. An analysis of genes indicated that some enzymes including PAL are considered to be involved in the main resistance processes in wheat [44]. Liu et al. [7] reported that the amount of *β*-1,3-glucanase was much higher in the incompatible interaction than that in the compatible interaction. In addition, a previous study showed that the resistant wheat contained higher activity of chitinase than the susceptible wheat [45,46]. In accordance with these reports, the present study found that the activities of four pathogenesis-related enzymes were significantly enhanced and higher in the incompatible system relative to the compatible system (Figure 5). This suggested that these disease resistance-related enzymes possibly contribute to greater disease resistance in the incompatible. As two important disease resistance genes in wheat, *TaNIT* and *TaLHY* may participate in regulating the defense response against stripe rust infection [47,48]. A previous study indicated that the expression of *TaLHY* was up-regulated after infection by CYR32 in Chuannong 19 with virus-induced gene silencing [48]. We showed that infection by *Pst* could result in the up-regulated expression of *TaNIT* and *TaLHY* in CM42 (Figure 6), which suggests that these two genes may be involved in the defense response to stripe rust in wheat. Furthermore, high expression of *TaNIT* and *TaLHY* in the incompatible indicated that these two genes play an important role in the disease resistance response at all stages in wheat.

Many research studies have shown that plant photosynthesis can be greatly reduced when disease symptoms are developing after inoculation with stripe rust [14,18,49,50]. Chlorophyll fluorescence has been shown to be a useful method for the investigation in photosynthesis under different stressful conditions including pathogen infection [51]. Our previous study indicated that a stronger decrease in PSII photochemistry occurred in the susceptible cultivar Sy95-71 compared with the resistant cultivar CN19 at 72 hpi. Similarly, the present study found that the compatible system showed a more significant decline in Fv/Fm, ΦPSII, and qP relative to the incompatible system at 120 hpi (Figure 7), which suggested a lower quantum yield of PSII in the compatible system. In contrast, the higher levels of NPQ in the compatible system indicated that excess light energy dissipated after infection by *Pst*. Furthermore, state transitions play an important role in the energy conserving response under environmental stresses. High fluorescence of state transitions in the compatible system at 120 hpi may be contributed to unbalance the complements of LCHII between PSI and PSII after infection by stripe rust. These findings were further confirmed by the results obtained from the photochemistry of PSI, which is usually shown to be seriously damaged under heavy environmental stresses [52,53,54]. In the present experiment, our results showed that PSI photochemistry significantly decreased after inoculation with *Pst* compared with the control, and the levels of PSI photochemistry in the compatible system were lower than that of the incompatible system at 48 and 120 hpi (Figure S5), which suggests that stripe rust infection resulted in the damage to PSI. In addition, several previous studies indicated that *P*n in *Pst*-infected wheat plants significantly decreased [50]. Chang et al. [49] also reported that the photosynthetic rate decreased in the compatible and incompatible systems at 120 hpi in wheat. Consistent with these reports, the present results showed that *Pst* infection markedly decreased *P*n and stomatal conductance, which suggests that pathogen infection may reduce photosynthetic performance in wheat. Moreover, the lower photosynthetic rate in the compatible system relative to the incompatible system was possible due to the damage of chlorophyll or the decline of green leaf areas [50]. Hence, these findings indicated that the incompatible system could alleviate wheat stripe rust damage to the photosynthetic apparatus more effectively than the compatible system.

Some previous studies have demonstrated that the PSII reaction center protein D1 is the key target site hampered by different environmental stresses [20,55]. Several previous studies indicated that stripe rust infection led to the decline in the content of D1 proteins in the susceptible wheat. Similarly, we found that only the amount of D1 was remarkably reduced in the compatible systems, which indicated that stripe rust infection mainly damaged the PSII reaction center in wheat plants. The more significant decline in D1 protein may be due to the severe damage to the PSII reaction center in the compatible system.

## 4. Materials and Methods

### 4.1. Plant Materials and Inoculation

Wheat cultivar Chuanmai42 (CM42) and two Chinese *Pst* pathotypes (CYR32 and V26) were used in this study. CM42 is a useful genetic resource for wheat production and breeding in Sichuan. It is highly susceptible to race V26 (IT = 4, compatible reaction), and highly resistant to race CYR32 (IT = 0, incompatible reaction) at all stages [8].

CM42 seeds with a uniform size were chosen and sterilized using 1% NaClO solution for 10 min. Then, these seeds were rinsed for several times and germinated for 3 days in Petri-dishes at room temperature in the dark. Wheat seedlings germinated uniformly were grown in a 20-cm diameter pot filled with organic soil and then put in the greenhouse with illumination of a 250 μmol photon m^−2^ s^−1^ for a 16-h photoperiod, at 16 °C, and 65% relative humidity. For the seedling stage inoculation, wheat seedlings were grown to the three-leaf stage. Inoculations were performed by inoculated freshly collected *Pst* urediniospores on the surfaces of the primary leaves with a paintbrush. Mock-inoculated plants were treated following the same way with sterile distilled water. To allow the fungus to infect the plants, these inoculated seedlings were placed with high humidity for 24 h at 10 °C in the dark and then moved to the greenhouse with 250 μmol photon m^−2^ s^−1^ under conditions of 14 h light/10 h darkness (16 °C/10 °C) and 75% relative humidity. Inoculated and control leaves were sampled at 0, 24, 48, and 120 h post inoculation (hpi) and immediately frozen in liquid nitrogen until use. The leaving plants were continually grown for observations of disease responses.

### 4.2. Chlorophyll Content, Total Protein Content, and Osmotic Regulators

Chlorophyll (Chl) content was measured as the method described previously [56]. The infected and non-infected leaves (0.5 g fresh weight) were ground in 80% (*v/v*) acetone at room temperature. After filtering, Chl content was measured at 663 and 645 nm with a spectrophotometer (Hitachi-U2000, Tokyo, Japan). The total soluble protein content was measured with the method of Lowry et al. [57] by using a spectrophotometer (Hitachi-U2000, Tokyo, Japan). Fresh leaves (0.5 g) were homogenized with 5 mL of sodium phosphate buffer (pH 7.2) and then supernatants were used for analyzing soluble protein after centrifugation (3000 rpm for 10 min at 4 °C). Soluble sugar was extracted with 80% ethanol solution in boiling water and its content was calculated after reacting with the anthrone reagent following the method of Thomas [58]. Proline was extracted in 3% (*w/v*) sulfosalicylic acid and its content was measured at 520 nm following the previous method [59].

### 4.3. Determinations of Lipid Peroxidation and Electrolyte Leakage

Lipid peroxidation was estimated based on the concentration of malondialdehyde (MDA), which was detected by the thiobarbituric acid (TBA) reaction as previously described by Luo et al. [60]. Fresh leaves (0.5 g) were homogenized in 5 mL of ice-cold 5% (*w/v*) trichloro acetic acid (TCA). After centrifugation (8,000 g for 10 min at 4 °C), 2 mL of the supernatant was mixed with 2 mL 5% TCA containing 0.67% TBA. The mixture was incubated at 95 °C for 0.5 h, then quickly cooled on ice, and centrifuged at 8000 g for 5 min. The concentration of MDA was calculated by measuring the difference of the absorbance of the supernatant at 532 and 600 nm using its extinction coefficient of 155 mM^−1^ cm^−1^. Electrolyte leakage of leaves was measured using a conductivity meter (DDSJ-308A, Shanghai Precision Instruments Co., Ltd., Shanghai, China) following the method of Chen et al. [20]. The conductivity of 95 °C for 20 min was normalized to 100%.

### 4.4. Assay of ROS and Tissue Staining

Accumulation of superoxide anion radicals (O_2_˙ˉ) and hydrogen peroxide (H_2_O_2_) in wheat leaves was observed by incubation with nitro blue tetrazolium (NBT, 0.5 mg mL^−1^) for 2 h and 3,3-diaminobenzidine (DAB, 2 mg mL^−1^) for 8 h, respectively. Then the stained samples were decolorized in glycerol: acetic acid: ethanol (1:1:3, *v/v/v*) in a boiling water bath for 0.5–2 h and photographed as described previously [14]. The contents of O_2_˙ˉ and H_2_O_2_ in leaves were determined according to the procedure of References [61] and [62], respectively. For the analysis of cell death, wheat leaves were stained by trypan blue solution (1.25 mg mL^−1^) and then cleared by chloral hydrate solution (2.5 g mL^−1^) following the previous method [63]. For the detection of callose accumulation, wheat leaves were stained by 0.01% (*w/v*) aniline blue solution for 15 min, according to the protocols of Shirasu et al. [64]. Fluorescent images were obtained with a fluorescence microscope (BX53 System, Olympus Corporation, Tokyo, Japan).

### 4.5. Antioxidant Systems

For determining antioxidant enzymes, fresh wheat leaves (0.5 g) were ground with 5 mL ice-cold 50 mM potassium phosphate buffer (pH 7.8) containing 2 mM ascorbate, 0.2 mM EDTA, and 2% (*w*/*v*) polyvinylpyrrolidone (PVP) at 4 °C. The supernatants were obtained after 20 min 12,000 g centrifugation at 4 °C. Superoxide dismutase (SOD), peroxidase (POD), catalase (CAT), ascorbate peroxidase (APX), glutathione reductase (GR), and glutathione peroxidase (GPX) activities were measured as previously described [65]. The contents of reduced ascorbic acid (AsA) and dehydroascorbate (DHA) were measured by using the HPLC method as described by Xu et al. [66]. The extraction and detection to reduced glutathione (GSH) and oxidized glutathione (GSSG) were performed, according to the procedure described by Bechtold et al. [67].

### 4.6. Measurements of Disease-Related Enzymes

For the phenylalanine ammonia lyase (PAL) assay, 0.5 g of fresh leaves were ground with ice-cold 0.1 M Tris-HCl buffer (pH 8.8) containing 1% PVP and 1 mM EDTA. After centrifugation (15,000 g for 10 min at 4 °C), the supernatant mixed with 50 mM Tris-HCl buffer (pH 8.8) and 20 mM L-phenylalanine was incubated for 30 min at 30 °C, and, subsequently, the reaction was stopped by adding 0.5 mL of 10% TCA. PAL activity was assayed according to the method described by Heide et al. [68]. For the polyphenol oxidase (PPO) assay, 0.5 g of fresh leaves were homogenized with 5 mL of 100 mM sodium phosphate buffer (pH 6.0) containing 0.1 mM EDTA on ice. The homogenate was centrifuged at 15,000 *g* for 5 min at 4 °C and then the extract was used for determining PPO activity following the method of Gao et al. [69]. A modified method was used to determine *β*-1,3-glucanase activity [70]. Fresh leaves (0.5 g) were ground in liquid nitrogen and mixed with 2 mL of 100 mM sodium acetate buffer (pH 5.5) containing 2 mM EDTA, 10 mM mercaptoethanol, and 2 mM phenylmethylsulfonylfluoride (PMSF). After centrifugation (10,000 *g* for 10 min at 4 °C), the supernatant was used as the crude enzyme for assay. Each reaction mixture contained 500 μL laminarin (2 mg mL^−1^), 480 μL 50 mM sodium acetate buffer (pH 4.5), and 20 μL enzyme extract. After incubation (37 °C, 10 min), 1 mL Somogyi’s reagent was added and the mixture was incubated at 100 °C for 10 min [71]. After cooling, 1 mL of Nelson’s reagent was added into the mixture [72]. Subsequently, the absorbance of the colored product was measured at 540 nm. Chitinase activity was measured colorimetrically using a modified method of van der Westhuizen et al. [73]. The absorbance at 585 nm was measured. The chitinase activity was calculated based on the standard curve related A_585nm_ to GlcNac concentration and expressed as μmol GlcNAc h^-1^ mg^-1^ protein.

### 4.7. Chlorophyll Fluorescence and State Transition

An imaging fluorometer (the Imaging PAM M-Series Chlorophyll Fluorescence System, Heinz-Walz Instruments, Effeltrich, Germany) was applied for measuring chlorophyll fluorescence following the manufacturer’s instructions at room temperature. All samples were kept in the dark for at least one hour before fluorescence measurements. A saturated pulse intensity of 8000 μmol m^−2^ s^−1^ and actinic light intensity of 1500 μmol m^−2^ s^−1^ were given in the present experiment. The maximum efficiency of PSII photochemistry (Fv/Fm), the non-photochemical quenching (NPQ), the quantum yield of PSII electron transport (ΦPSII), and the photochemical quenching (qP) were calculated following the equations of Maxwell and Johnson [74]. Chl *a* fluorescence and the state of PSI photochemistry in un-inoculated and inoculated leaves were obtained using the Dual-PAM-100 fluorometer (Heinz-Walz Instruments, Effeltrich, Germany) at room temperature following the previous method [75]. The effective quantum yield of PSI (Φ_PSI_), oxidation status of the PSI donor side (Φ_NA_), and a reduction status of the PSI acceptor side (Φ_ND_) were calculated as the previous method [76]. State transition was measured with Dual-PAM-100 fluorometer in intact and dark-adapted leaves, according to the established method [77]. The maximal PSII fluorescence (*F*_m_) was recorded before triggering a condition of a state transition. Wheat seedlings were subjected to red (635 nm) and far red light (720 nm) using LED light sources (SL 3500-R-D). The maximal fluorescence in state 1 (*F*_m1_) and state 2 (*F*_m2_) were recorded at the end of each state transition cycle by the application of the saturating light pulse (4000 μmol photons m^−2^ s^−1^).

### 4.8. Gas Exchange Measurements

An open GSF-3000 photosynthesis system (Heinz-Walz Instruments, Effeltrich, Germany) was used to measure a net photosynthetic rate, a transpiration rate, intercellular CO_2_ concentration, and stomatal conductance. The 360 μmol mol^−1^ CO_2_ concentration, 60%–80% relative humidity, and a photosynthetically active radiation (PAR) of 1500 μmol photons m^−2^ s^−1^ was set for measurement of the CO_2_ assimilation rate at room temperature (25 °C) [78].

### 4.9. Immunoblotting Analysis of Thylakoid Membrane Proteins

The isolation of thylakoid membrane proteins from un-inoculated and inoculated leaves was carried out under dim light, according to the procedure described by Chen et al. [79]. Separation of thylakoid membrane proteins were performed by SDS-PAGE (6% acrylamide stacking gel + 15% separation gel + 6 M urea). For Western blotting, proteins were shifted to a polyvinylidene fluoride (PVDF) membrane (Immobilone, Millipore, Darmstadt, Germany) and, subsequently, 5% skimmed milk was used to block the membrane. The transferred proteins were then detected with protein-specific antibodies against representative PSI proteins (Lhca1, Lhca2, Lhca3, Lhca4, and PsaD) and PSII proteins (D1, D2, CP43, Lhcb1, Lhcb2, Lhcb3, Lhcb4, Lhcb5, and Lhcb6), which were purchased from Agrisera (Umea, Sweden). To detect the immunoblotting signals, the horseradish peroxidase-conjugated secondary antibody (Agrisera Comp., Umea, Sweden) and a chemiluminescent detection system (ECL, GE Healthcare, Buckinghamshire, UK) were applied. 

### 4.10. Quantitative Real-Time PCR (qRT-PCR) 

Total RNA from wheat seedlings was isolated using the Plant RNA Isolation Kit (Invitrogen, Carlsbad, CA, USA) and then treated with DNase I (Code No. 2212, Takara) for removal of genomic DNA, according to the manufacturer’s instructions. qRT-PCR was applied to measure the expression levels of *TaLHY* (late elongated hypocotyl) and *TaNIT* (nitrilase). The following primers were used, forward 5’-CCTACTGCTTCCTTTCCCACAAC-3’ and reverse 5’-CTCTCCTTTTCCACTCTCGTCTG-3’ for *TaLHY*, forward 5’-CGACTACCTACGCAGGAAGCAC-3’ and reverse 5’-CTAACCAGCATCTTCTCCGAA-3’ for *TaNIT*. All reactions were set up as previously described by Zhang et al. [48]. The SYBR *Premix Ex Taq*^TM^ II Master (TaKaRa, Japan) was used for quantitative assays of gene expression. The relative expression of the gene *TaNIT* and *TaLHY* was quantified using the 2^-ΔΔ*C*t^ method [80]. The expression of *TaActin* gene was used as an internal control to normalize all data.

### 4.11. Statistical Analysis 

All analyses were done in three to four independent biological replicates and values were presented with mean ± standard deviation (SD). Data management and statistical analysis were carried out using SPSS 19.0 statistical software (IBM, Chicago, IL, USA). The means were compared using Duncan’s multiplication range test. A different letter was marked above the error bar to show a significant difference at the level of *p* < 0.05.

## 5. Conclusions

In the present study, our results showed that stripe rust infection resulted in the clear ROS accumulation and the decline in photosynthetic efficiency in the compatible and incompatible systems. The activities of four disease resistance-related enzymes and the expression of two disease-resistant genes were significantly up-regulated after inoculation with stripe rust. In addition, we found that the incompatible system may be more effective in alleviating ROS toxicity via the antioxidant system and maintain high photosynthetic capacity by alleviating the oxidative damage to the PSII reaction center. Therefore, we propose that the antioxidant defense systems, disease resistance-related enzymes, and disease resistance genes likely play the key roles in stripe rust resistance in wheat.

## Supplementary Materials

Supplementary materials can be found at https://www.mdpi.com/1422-0067/21/1/268/s1.
