# Supplementary material for: Effects of Stripe Rust Infection on the Levels of Redox Balance and Photosynthetic Capacities in Wheat
Source: Int J Mol Sci. 2019 Dec 31;21(1):268. doi: 10.3390/ijms21010268 (PMC6981720; doi:10.3390/ijms21010268)
Supplement: Supplementary file 1 [file ijms-21-00268-s001.pdf]

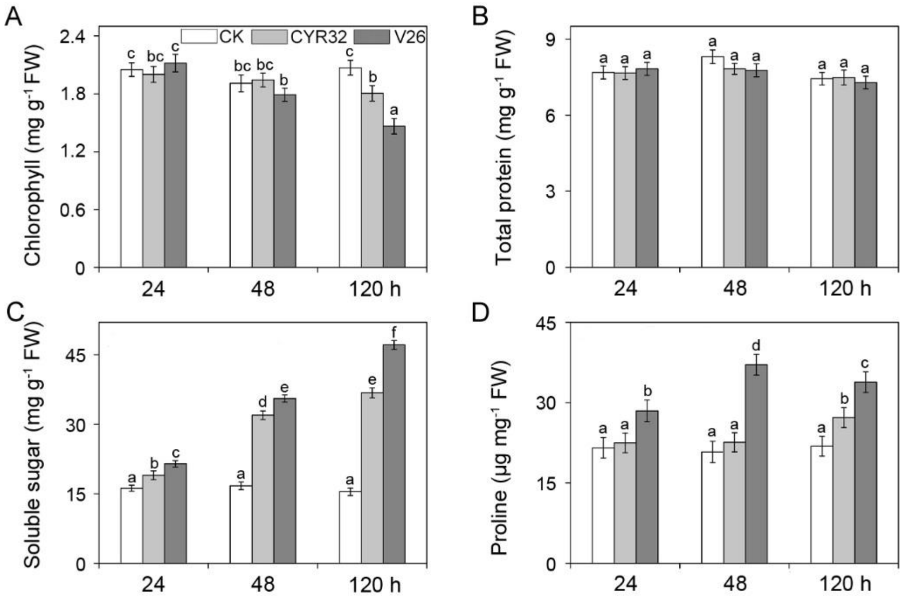

Figure S1. Chlorophyll content (**A**), total protein content (**B**), soluble sugar (**C**), and proline (**D**) in wheat cultivar CM42 inoculated with CYR32 and V26. Bars represent standard deviations (SD) which were calculated from four independent biological replicates (*n* = 4). Different letters note significant differences among different treatments ($P < 0.05$) following Duncan's multiplication range test. CK, un-inoculated wheat plants. 24-120 hpi represent 24, 48, and 120 h post-inoculation.

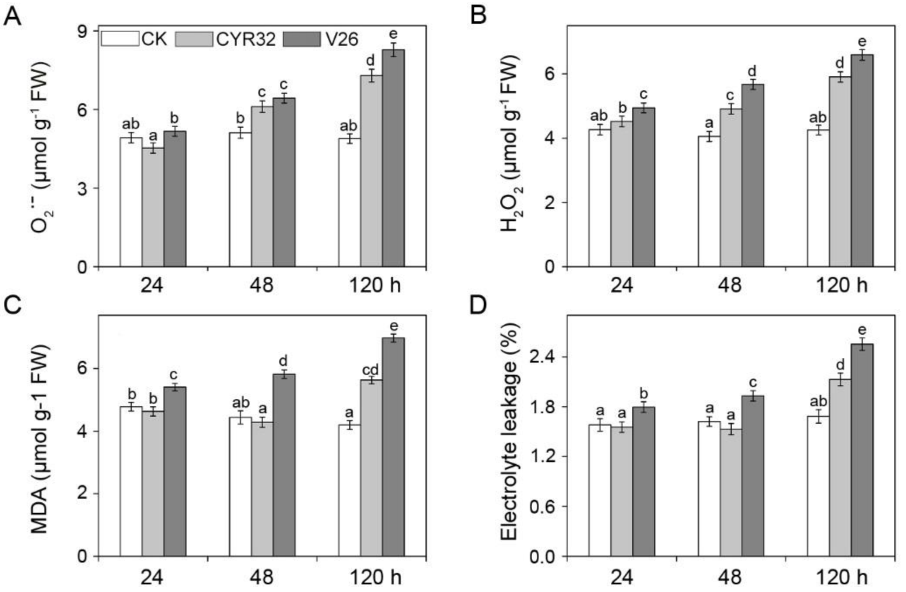

Figure S2. Superoxide anion radicals ($O_2^{\cdot-}$) production rate (**A**), hydrogen peroxide (H_2O_2) content (**B**), the malondialdehyde (MDA) content (**C**), and electrolyte leakage (**D**) in wheat cultivar CM42

inoculated with CYR32 and V26. Bars represent standard deviations (SD) which were calculated from four independent biological replicates (n = 4). Different letters note significant differences among different treatments ($P < 0.05$) following Duncan's multiplication range test. CK, un-inoculated wheat plants. 24-120 hpi represent 24, 48, and 120 h post-inoculation.

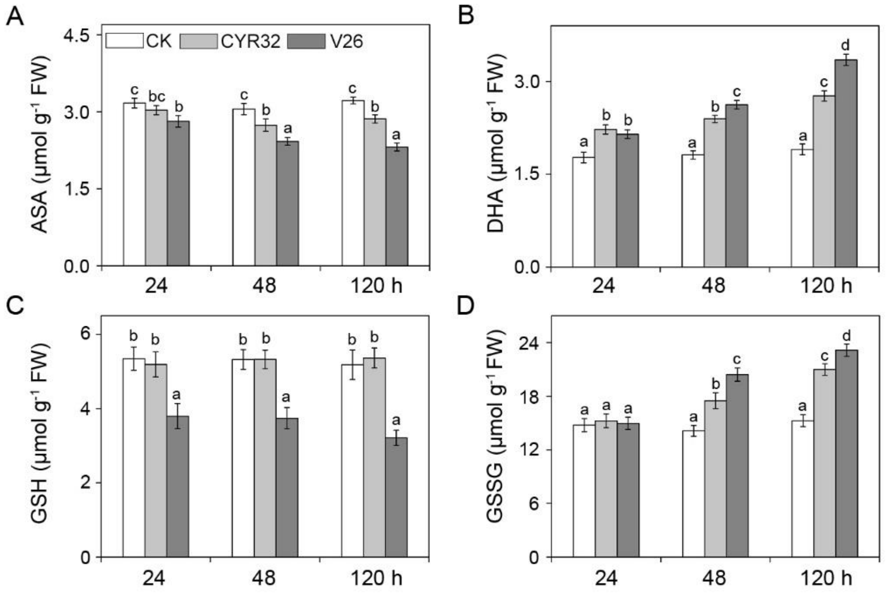

Figure S3. The concentrations of antioxidants in wheat cultivar CM42 inoculated with CYR32 and V26. AsA, ascorbic acid (**A**); DHA, dehydroascorbic acid (**B**); GSH, reduced glutathione (**C**); GSSG, oxidized glutathione (**D**). Bars represent standard deviations (SD) which were calculated from four independent biological replicates (n = 4). Different letters note significant differences among different treatments ($P < 0.05$) following Duncan's multiplication range test. CK, un-inoculated wheat plants. 24-120 hpi represent 24, 48, and 120 h post-inoculation.

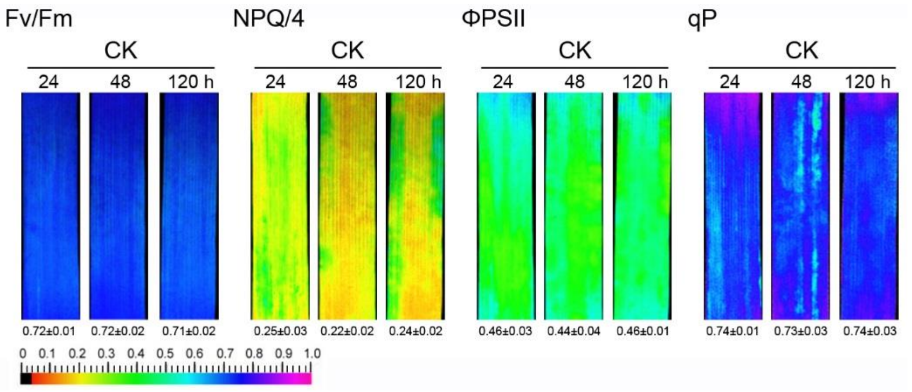

Figure S4. Chlorophyll fluorescence parameters (Fv/Fm, maximum efficiency of PSII photochemistry; NPQ, non-photochemical quenching; ΦPSII, quantum yield of PSII electron transport; qP, photochemical quenching) in the un-inoculated wheat plants at 24, 48, and 120 hpi. Quantitative values (± SD) are shown below each fluorescence images. CK, un-inoculated wheat plants.

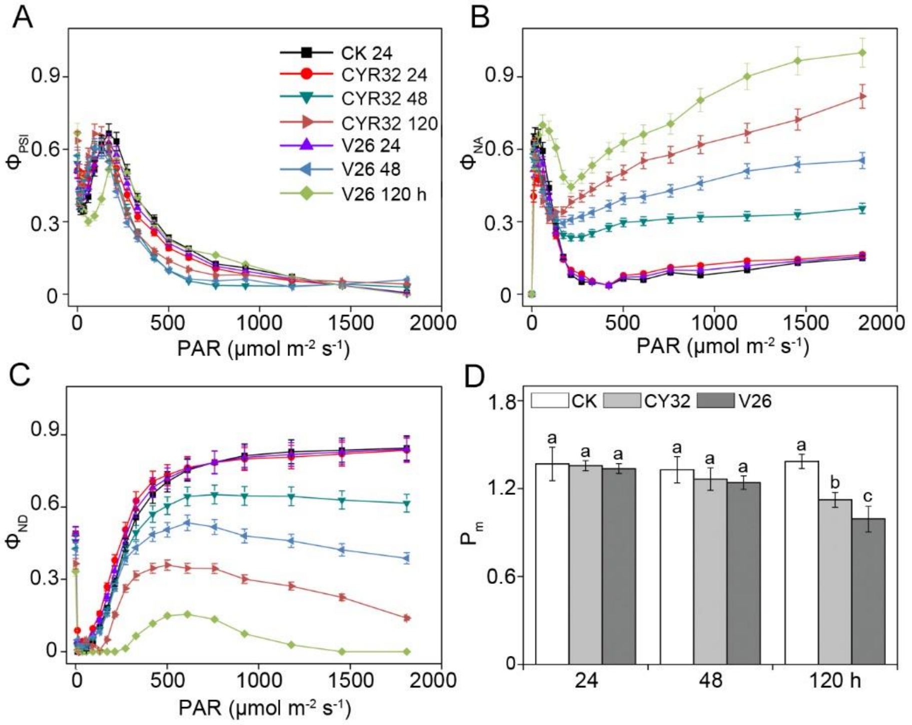

Figure S5. The values of parameters derived from P700 absorbance in wheat cultivar CM42 inoculated with CYR32 and V26. Φ_{PSI} (**A**), effective quantum yield of photosystem I; Φ_{NA} (**B**), quantum yield of non-photochemical energy dissipation of PSI reaction centers due to acceptor side

limitation; Φ_{ND} (**C**), quantum yield of nonphotochemical energy dissipation in PSI reaction centers due to donor side limitation; *P*m (**D**), maximal P700 signal. Bars represent standard deviations (SD) which were calculated from four independent biological replicates (*n* = 4). Different letters note significant differences among different treatments ($P < 0.05$) following Duncan's multiplication range test. CK, un-inoculated wheat plants. 24-120 hpi represent 24, 48, and 120 h post-inoculation.

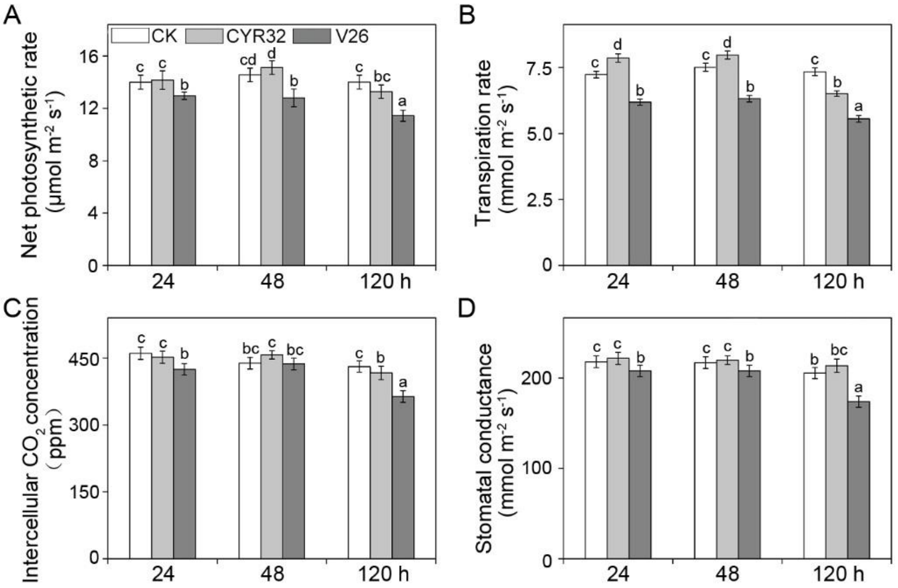

Figure S6. Net photosynthetic rate (**A**), transpiration rate (**B**), intercellular CO_2 concentration (**C**), and stomatal conductance (**D**) in wheat cultivar CM42 inoculated with CYR32 and V26. Bars represent standard deviations (SD) which were calculated from four independent biological replicates (*n* = 4). Different letters note significant differences among different treatments ($P < 0.05$) following Duncan's multiplication range test. CK, un-inoculated wheat plants. 24-120 hpi represent 24, 48, and 120 h post-inoculation.
